# Supplementary material for: Sustained-input switches for transcription factors and microRNAs are central building blocks of eukaryotic gene circuits
Source: Genome Biol. 2013 Aug 23;14(8):R85. doi: 10.1186/gb-2013-14-8-r85 (PMC4054853; doi:10.1186/gb-2013-14-8-r85)
Supplement: Additional file 5 — HTML Browsable Motif Output. Zipped folder containing all WaRSwap and FANMOD motif output, viewable in a web browser. [file gb-2013-14-8-r85-S5.ZIP › HTML_browsable_motif_output/FANMOD_ath_tair9/sigs_fanmodm-2000.pvals.heatmaps.html/motif_id_6_000101002_tftype_ath_upstream_-1000_0.html]

```
BG_MODEL = FANMOD
MOTIF_ID = 6_000101002
TF_TYPE = ath
UPSTREAM = -1000_0


PVals
FN_0.2	FN_0.4	FN_0.6	FN_0.8
dg_60.genes	0.501	0.696	0.521	0
dg_70.genes	0.508	0.696	0.516	0
dg_80.genes	0.524	0.69	0.543	0

ZScores
FN_0.2	FN_0.4	FN_0.6	FN_0.8
dg_60.genes	-0.072	-0.545	-0.049	2.533
dg_70.genes	-0.079	-0.516	-0.048	2.531
dg_80.genes	-0.07	-0.54	-0.09	2.485

StDevs
FN_0.2	FN_0.4	FN_0.6	FN_0.8
dg_60.genes	35981.891	33399.601	12511.988	1974.812
dg_70.genes	35683.849	32329.201	12336.443	1953.351
dg_80.genes	36550.621	32932.714	12540.947	2045.599
```
